# The Management of Cyanotic Spells in Children with Oesophageal Atresia

**DOI:** 10.3389/fped.2017.00106

**Published:** 2017-05-15

**Authors:** Mathieu Bergeron, Aliza P. Cohen, Robin T. Cotton

**Affiliations:** ^1^Division of Pediatric Otolaryngology–Head and Neck Surgery, Cincinnati Children’s Hospital Medical Center, Cincinnati, OH, USA; ^2^Department of Otolaryngology–Head and Neck Surgery, University of Cincinnati College of Medicine, Cincinnati, OH, USA

**Keywords:** oesophageal atresia, oesophageal atresia/tracheoesophageal fistula, cyanosis, pediatric, other, tracheomalacia

## Abstract

Cyanotic spells, also known as blue spells, dying spells, or apparent life-threatening events, refer to a bluish tone visible in the mucosal membranes and skin caused by an oxygen decrease in the peripheral circulation. Although this decrease may be transient and benign, it may also be indicative of a severe underlying problem that requires immediate intervention. Children with oesophageal atresia (OA) are at risk for a number of coexisting conditions that may trigger cyanotic spells. This current article will focus on the management of cyanotic spells both in children with innominate artery compression and those with tracheomalacia.

## Introduction

Cyanotic spells, also known as blue spells, dying spells, or apparent life-threatening events, refer to a bluish tone visible in the mucosal membranes and skin caused by an oxygen decrease in the peripheral circulation. Although this decrease may be transient and benign, it may also be indicative of a severe underlying problem that requires immediate intervention. If a high level of vigilance is not maintained and appropriate measures are not taken in a timely manner when cyanotic spells are severe and prolonged, they may lead to bradycardia, cardiorespiratory arrest, and ultimately death.

Children with oesophageal atresia (OA) are at risk for a number of coexisting conditions that may trigger cyanotic spells. In an early study (1963), Fearon and Shortreed (1) described episodic reflex apnea as the physiologic mechanism thought to cause these spells and maintained that they were sometimes associated with tracheobronchial compression secondary to a double aortic arch or an anomalous innominate artery. They presumed that reflex apnea was triggered when vagal afferent nerve fibers were stimulated. They also believed that swallowing and other forms of transient intrathoracic pressure increase could trigger a reflex type respiratory arrest (2).

The association between OA and cyanotic spells in children with tracheomalacia has also been recognized for the past several decades (3, 4). Children with OA are at risk for various comorbidities in addition to either innominate artery compression or tracheomalacia. They are prone to a number of conditions such as oesophageal dysmotility with slow transit and risk of bolus obstruction, gastro-esophageal reflux, aspiration, risk of anastomotic strictures and proximal dilatation of the oesophageal and pouch, and diverticulum. They also have high incidence of concurrent airway pathology such as laryngeal cleft and vocal cord paralysis (5).

In view of the well-established clinical associations between OA and both innominate artery compression and tracheomalacia, our discussion will focus on the management of cyanotic spells in children with these conditions.

## Innominate Artery Compression

Gross and Neuhauser were the first group to describe anterior tracheal compression by an innominate artery arising on the left side of the trachea (6). The innominate artery normally crosses the anterior aspect of the trachea from left to right about 1 cm above the carina (7, 8). When it arises more downstream along the aorta on the left, it may lead to anterior compression of the tracheal cartilages. Three other explanations (9) accounting for tracheal compression have also been described. The innominate artery may be tauter than normal, leading to compression of the trachea. Also, tracheal cartilages may be unusually compliant and more easily compressed. Finally, dilation of other structures such as the heart, esophagus, or thymus can cause mediastinal crowding, thus causing the innominate artery to compress the trachea (9). In all of these clinical scenarios, the innominate artery creates an indentation in the trachea. Although the indentation is visualized endoscopically in many children, it is clinically important only when it significantly compresses the trachea (2).

Symptoms related to innominate artery compression appear during the early months of life and may range from exceedingly subtle to obvious. In the former clinical setting, the problem may be misdiagnosed and treated for years as resilient asthma or croup. Recurrent pneumonia and bronchitis are also a possible presentation of innominate artery compression. These patients are prone to recurrent chest infection because they have difficulty in passing secretions through the narrowed segment of the trachea (10) Moreover, during coughing, the tracheal lumen may be collapsed at this site, leading to further entrapment of secretions (10). Cyanotic episodes generally begin after 2 to 3 months of age and characteristically occur during or after a meal or during coughing or crying (3, 11, 12). During feeding, bolus transfer compresses the posterior wall of the trachea at the malacic site (13). The most dramatic consequence is cardiorespiratory arrest (3, 4). These events are often unpredictable and can occur without other respiratory symptoms.

## Tracheomalacia

Tracheomalacia (either primary or secondary) is the most common congenital tracheal anomaly, and most children are either asymptomatic or minimally symptomatic. This anomaly is characterized by an abnormally compliant trachea displaying dynamic collapse during the respiratory cycle or when coughing. Either the entire trachea or specific portions of the trachea (i.e., the anterior and/or posterior walls) can be involved. Tracheoesophageal fistula (TOF) is a commonly associated abnormality (4).

Although tracheomalacia can either be the result of abnormal embryologic development of the trachea or occur following the repair of OA and TOF, these two etiologies are not mutually exclusive and may coexist. When the cause is primarily embryologic, it is the result of a disproportion between the cartilaginous and membranous components of the trachea. When tracheomalacia is observed in children with OA after TOF repair, the trachea retains an abnormal configuration, with a wide membranous portion rather than the normal C-shape (12). This predisposes to collapse. The interval between OA repair and the appearance of respiratory symptoms could be less than 30 days (8). As with vascular compression, these symptoms may range from exceedingly subtle to obvious; in the former clinical scenario, it can be misdiagnosed and treated for years as resilient asthma or croup (Table 1).

**Table 1:** **Symptoms of tracheomalacia**.

Asymptomatic Dyspnea (at rest or with exertion) Cough (brassy type) Sputum retention Wheezing/stridor Recurrent pulmonary infection Bronchitis Cyanotic spells

There is no accepted classification of tracheomalacia; however, classifying this anomaly as mild, moderate, or severe assists with clinical management. Mild tracheomalacia is characterized by respiratory difficulties associated with infectious processes such as croup or bronchiolitis. Moderate tracheomalacia typically presents with stridor, wheezing, recurrent respiratory infections, and cyanosis associated with infection. Severe tracheomalacia is characterized by upper airway obstruction, trapping of secretions with pulmonary infection, cyanotic spells, and sometimes death. Shah et al. reported that patients with severe tracheomalacia were significantly more likely to experience cyanotic spells, with an odds ratio of 180 (14). Although mild tracheomalacia is watched expectantly and anticipated to improve with time (15), more severe symptoms warrant intervention. When cyanotic spells occur, prompt intervention is essential. Events such as infections, general anesthesia, or extubation may precipitate and exacerbate symptoms (16, 17).

Cyanotic episodes generally begin after 2–3 months of age and characteristically occur during or after a meal or during coughing or crying (4, 12). Feeding and coughing increase intrathoracic pressure, leading to further tracheal compression (12). As in children with innominate artery compression, the most dramatic consequence of cyanotic spells in children with severe tracheomalacia is cardiorespiratory arrest (4, 12).

## Oesophageal Atresia and Tracheoesophageal Fistula

Respiratory symptoms are common in patients with repaired OA and TOF, and tracheomalacia that precipitates life-threatening cyanotic spells has been reported to be the most frequent serious problem following OA and TOF repair (4). It is, however, difficult to distinguish between symptoms caused by tracheomalacia and those caused by problems such as recurrent pneumonia and aspiration (18). Although tracheomalacia is reported to be present in 75–90% of pathologic specimens, it is clinically significant in only 10–30% (19, 20), with the lower half of the trachea being affected in the region of the TOF, most likely due to the malformation and deficiency of the tracheal wall at that site (21). Patients with OA without TOF often have a normal trachea and no airway symptoms (22).

## Diagnostic Evaluation

**Figure 1:**
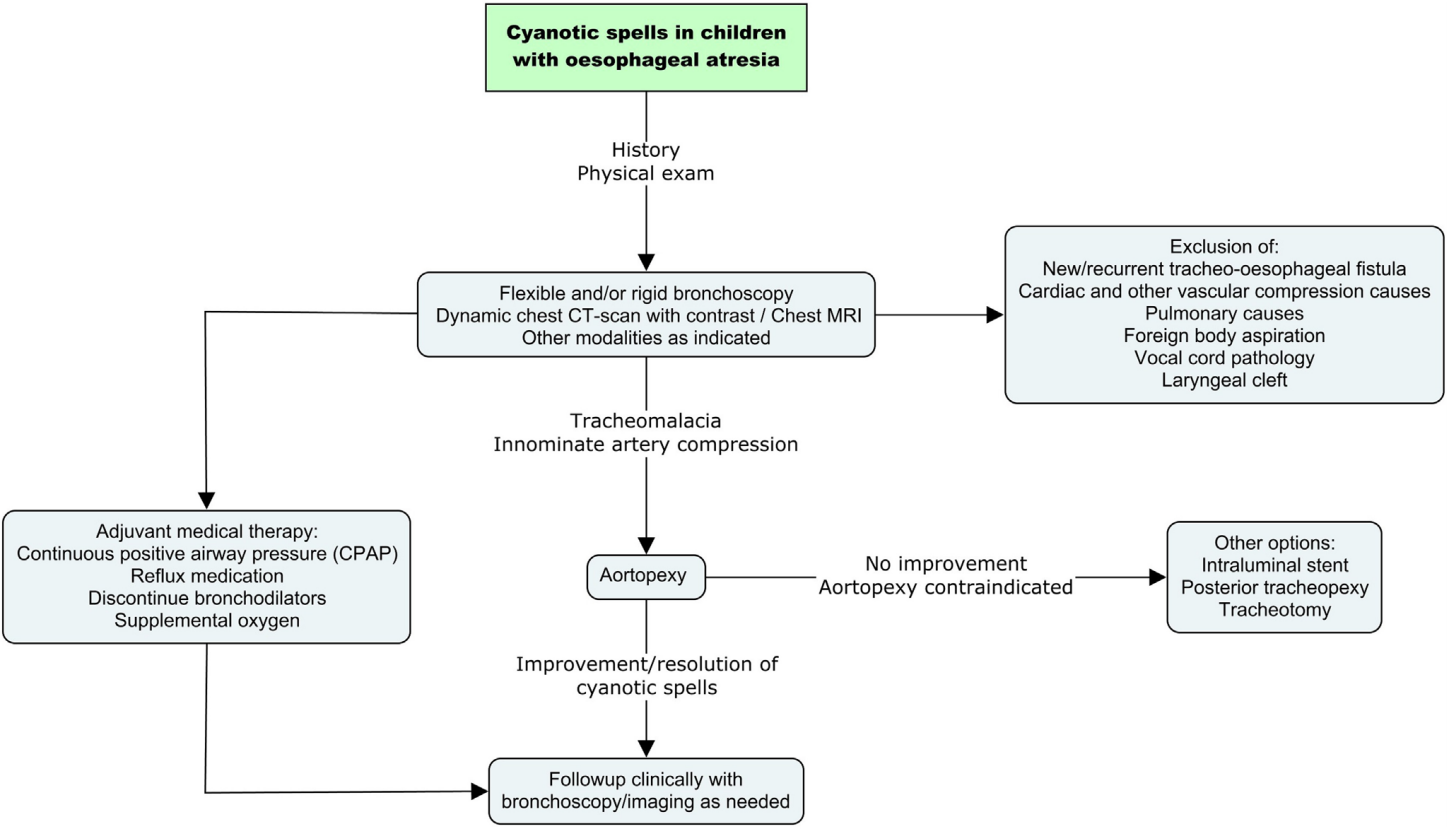
**The management of cyanotic spells in children with oesophageal atresia**.

All children with OA who have a cyanotic spell require prompt diagnostic assessment (Figure 1). Initially, it is essential to rule out a missed or recurrent TOF. The latter occurs in approximately 10% of patients (21). Other airway conditions such as laryngeal cleft, vocal cord pathology, tracheal diverticulum/pouch should be evaluated and treated accordingly. Flexible or rigid bronchoscopy is the gold standard of investigations. Bronchoscopy provides valuable information regarding vascular compression and tracheal collapse. It is performed under light general anesthesia with the child spontaneously ventilating.

**Figure 2:**
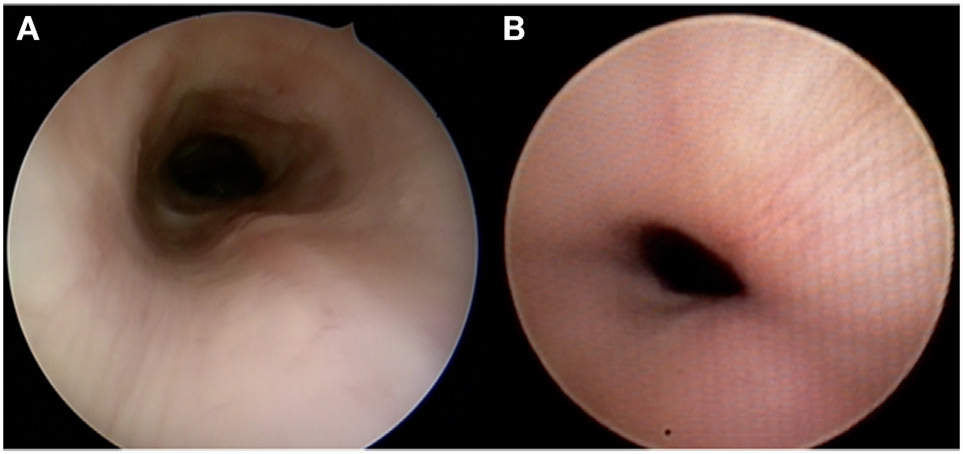
**Different appearances of tracheomalacia as seen with rigid bronchoscopy using a telescope only (A) and flexible bronchoscopy (B)**.

Flexible and rigid bronchoscopy are complementary diagnostic tools, with each having advantages and disadvantages (Table 2). At Cincinnati Children’s Hospital, experts on the pulmonary and otolaryngology team evaluate the airway with both flexible and rigid bronchoscopes. We perform rigid endoscopy with a telescope only rather than through a rigid ventilating bronchoscope to lessen distortion of anatomy. Flexible bronchoscopy requires less sedation than rigid endoscopy and allows clinicians to appreciate dynamic collapse while the child is spontaneously breathing. In smaller or less specialized pediatric centers, rigid bronchoscopy may be more readily available and is therefore a widely used alternative. The rigid telescope is often less obstructive than the flexible scope and may be better tolerated by the patient; however, it requires a deeper level of anesthesia and may falsely stent the anterior or posterior tracheal wall, thus obscuring visualization (Figure 2). In both rigid and flexible endoscopic approaches, adequate anesthesia is crucial. If anesthesia is too light, it may induce vigorous respiratory efforts and cause more pronounced malacia. If anesthesia is too heavy, it may mask malacia because the patient is not breathing independently, thus limiting the extent of dynamic collapse. Tracheomalacia can also be masked by positive pressure during insufflation. When performing an endoscopy, the clinician should estimate the percentage of airway collapse. Generally, an anteroposterior collapse of 75% with a cough or expiration is considered to be severe (4). In children with innominate artery or other vascular compression, anterior or anterolateral extrinsic pulsatile compression of the airway can be visualized (9).

**Table 2:** **Confounding factors associated with the bronchoscopic assessment of tracheomalacia**.

Factors that underestimate the severity of tracheomalacia	Stenting effect of rigid bronchoscopy Positive pressure Paralytic agents Patient is too heavily sedated
Factors that overestimate the severity of tracheomalacia	Engaging the suction channel during flexible bronchoscopy Patient is too lightly sedated

Imaging studies provide useful information regarding tracheal anatomy and compression of the trachea. High-resolution chest computed tomography (CT) with contrast and magnetic resonance imaging (MRI) are valuable in delineating innominate artery or other vascular compression or cartilaginous anomaly (23). When vascular compression is suspected, the imaging test of choice is MRI, as it also better demonstrates mediastinal vasculature and lower airway anatomy (9). Chest CT can be combined with 3D reconstruction and unlike MRI may not require sedation to acquire the images if the child is compliant. This allows exploration of regions inaccessible with endoscopy, such as areas distal to severe luminal obstruction. Similar to endoscopy, CT and MRI images are highly dependent on the level of sedation and respiratory effort of the patient.

Dynamic CT scan is a newer modality that provides dynamic images of the airway during the respiratory cycle. Images are taken when the patient is breathing or coughing. The patency of the lumen of the tracheobronchial tree is evaluated. The normal decrease in caliber during tidal breathing in children is around 30%. A collapse ranging from 30 to 75% may be monitored, depending on the severity of clinical symptoms (24). A collapse of more than 75% is likely to be considered significant and may require intervention (24). Dynamic CT can be performed without sedation and without contrast; the radiation dose is equivalent to that associated with a high resolution CT scan.

Airway fluoroscopy has also long been used to diagnose vascular compression and tracheomalacia, and it remains an alternative when the previously discussed diagnostic modalities are not available. However, a small study (*n* = 22) published in 2012 demonstrated that fluoroscopy has poor sensitivity (23.8%) but high specificity (100%) when used as a diagnostic tool for tracheomalacia (25). It is primarily used to diagnose OA or TOF. To assess these two conditions, fluoroscopy and/or oesophagogastroscopy could provide valuable information along the other previously described investigations about the site of the fistula and the extent of the atresia (26). Complete esophageal investigation could also include manometry and pH monitoring when judged relevant (26, 27).

A chest X-ray or high-resolution CT of the chest can be performed to assess the impact of recurrent infections, chronic aspiration, or the possible presence of a mediastinal mass. Pulmonary function testing (PFT) is used to show a reduction in peak expiratory flow and is typically abnormal in patients with tracheomalacia or innominate arterial compression; nevertheless, this is not a finding specific to tracheomalacia or innominate artery compression. PFT can be difficult to perform and interpret in young children who are uncooperative and has a positive predictive value of only 74% for tracheomalacia (9). Overnight polysomnography may be useful to quantify the impact of the obstruction and to plan decannulation for children in whom a tracheotomy has been placed (28).

## Management

### Cyanotic Spells Secondary to Innominate Artery or Other Vascular Compression

When cyanotic spells are secondary to innominate artery or other vascular compression, a number of medical and surgical management options are currently used. Prior to surgical management, children with cyanotic spells must be optimized. Pneumonia and bronchitis should be managed with a course of antibiotics. If necessary, supportive continuous positive airway pressure (CPAP) and oxygen should also be used.

**Figure 3:**
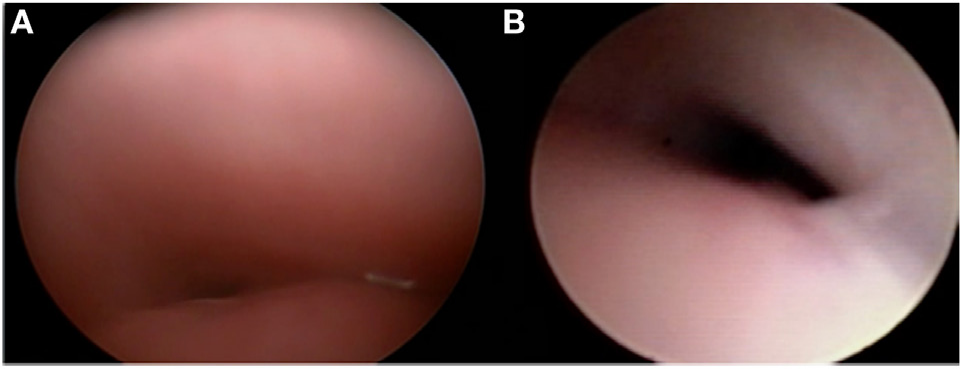
**Tracheomalacia before (A) and after aortopexy (B)**.

The mainstay of surgical management is aortopexy (29). In the setting of anterior wall tracheal collapse, this procedure can be performed either thoracoscopically or by using an open approach (Figure 3). The goal of this operation is to treat the airway collapse by ventral suspension of the trachea (30). Sutures are placed in the pericardial reflection over the aortic root and in the adventitia of the aortic arch and then tied to the underside of the sternum. As the aorta is pulled forward, fibrous attachments between the aorta and the trachea ensure that the front wall of the trachea is pulled forward, opening the lumen. Preoperative imaging enables the clinician to evaluate the size of the thymus and determine the amount of space available to move the aorta forward. When performed by thoracoscopy or left lateral thoracotomy, the surgery consists of thymectomy and subsequent minimal dissection of the lateral and posterior aorta prior to suspending the aorta to the posterior sternum. Intraoperative endoscopy is valuable and allows the surgeon to note improvement of the airway lumen. However, the endoscopic appearance of the trachea may not immediately change and the trachea may appear to be relatively malacic even several months after surgery (31). Complete response rates vary; however, they are reportedly as high as high as 100%, depending on the definition of response (32).

Although both reimplantation of the innominate artery and ligation and division of the innominate artery have historically been described, they are rarely performed. Literature pertaining to these procedures is scant, and success rates are not as high as those achieved with aortopexy (29). Management of other vascular compression such as double aortic arch and vascular ring require specific treatment. This includes various surgical techniques, primarily surgical division of these anomalies (33). The placement of intratracheal stents is not advised.

### Cyanotic Spells Secondary to Tracheomalacia

Once a bronchoscopic assessment has been performed, the clinician should know the severity and location of the malacia, particularly the possible presence of associated bronchomalacia, and be able to determine whether positive pressure support improves the malacia (34).

The same preoperative medical options described for innominate artery compression are used to address cyanotic spells in children with tracheomalacia. It is, however, important to note that management is symptom driven, not based on bronchoscopic appearance (34). When CPAP is specifically indicated, a tracheostomy may be required. Bronchodilators are not indicated, as they decrease posterior wall rigidity and may exacerbate tracheomalacia. If used, however, they must be discontinued prior to surgery. The use of airway smooth muscle stimulants such as bethanechol are sometimes helpful (35). Gastroesophageal reflux is commonly seen in children with tracheomalacia. Treatment with a proton pump inhibitor is an essential part of preoperative optimization (36). Other options include H2 antagonists, low-dose erythromycin, and fundoplication for refractory cases (37).

Surgical therapy depends on the area affected (anterior and/or posterior wall) and its location (extrathoracic trachea versus intrathoracic trachea). Surgical interventions that should be considered include aortopexy, intraluminal stenting, tracheotomy, posterior tracheopexy, external scaffold, and cartilage grafts. Regardless of the intervention, parents should be made aware of the possibility that surgery may not result in a complete response. For those cases, parents should undergo further counseling and basic life support training to decrease anxiety. Moreover, children may benefit from various interventions, such as texture modification with pacing and proper positioning during feeding, use of annual influenza vaccination, chest physiotherapy, reduction in childcare attendance, no exposure to passive smoking, and maximization of anti-reflux measures including fundoplication if appropriate (38).

### Aortopexy

Aortopexy (described above) represents the first line of treatment. In the case of anterior wall tracheal collapse, this procedure can be performed either thoracoscopically or by using an open approach. It is ideally suited for children with isolated symptomatic tracheomalacia that is most severe in the mid-tracheal region. It is not appropriate if severe bronchomalacia coexists, as it would not resolve distal collapse. Preoperative imaging allows evaluation of the size of the thymus and determination of the extent of space to move the aorta forward. When performed by thoracoscopy or left lateral thoracotomy, the surgery consists of thymectomy with subsequent minimal dissection of lateral and posterior aorta prior to suspending the aorta to the posterior sternum. Intraoperative endoscopy is valuable and allows the surgeon to visualize improvement of the airway lumen. However, as stated previously, the endoscopic appearance of the trachea may not change immediately. Postoperative recurrence rates necessitating revision range from 10 to 25% (30). Overall, few complications have been reported and the operation is usually well tolerated.

### Intraluminal Stenting

Intraluminal stenting for tracheomalacia should be reserved for highly selective cases (39) and is considered a last resort under special circumstances. This approach should be avoided in children with tracheomalacia and concurrent bronchomalacia since stenting of the distal bronchus is difficult and rarely successful. Stenting can be either temporary or permanent and a large variety of stents exist. Intratracheal metal stents may be an appropriate choice as a temporizing measure in that they expand enough to grip but not integrate with the mucosa. Ideally, they should be removed within a few weeks. Silicone stents are also a temporary measure and their use is limited to older children. There are reports of migration if not secured appropriately, creation of intraluminal biofilm leading to infection, and granulation tissue at either end of the stent (40, 41). If available, biodegradable airway stents may offer a better option, as they do not require removal and spontaneously disintegrate with time. Although data regarding their use are scant, no major complications have been reported to date (42).

### Tracheotomy

Although tracheotomy is not curative and has inherent risks in a child, it may be used as a temporizing measure with the expectation that improvement will occur over time. When a tracheotomy is placed, the customized inner shaft length must bypass the malacic segment of the trachea; this sometimes requires a cannula that is specifically fashioned for an individual patient. If necessary, CPAP and oxygen can be provided through the cannula. In older children in whom the tracheomalacia has improved, it generally takes longer to wean and decannulate than in children without a history of malacia. Moreover, the tracheotomy itself may create an area of tracheomalacia and suprastomal collapse by weakening the cartilage where it was previously inserted.

### Other Options and Experimental Modalities

**Figure 4:**
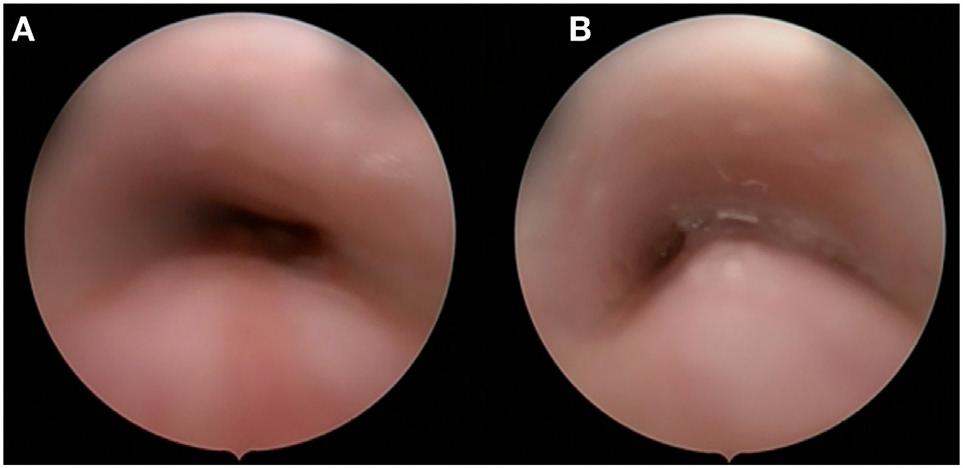
**Rigid endoscopy with telescope only at inspiration (A) and expiration (B)**.

Posterior tracheopexy, a technique developed at Boston Children’s Hospital, has been reported to stabilize the posterior membrane (43). This procedure can be performed with aortopexy to synergistically relieve anterior tracheal compression. In children with significant tracheomalacia associated with OA and/or TOF, the main component of the airway collapse is often the posterior tracheal membrane causing the posterior trachea to protrude into the tracheal lumen during exhalations—thus resulting in airway obstruction (Figure 4) (44). This technique can be performed concurrent with esophageal surgery.

Although extratracheal scaffold stents have been studied, they are rarely used because they will not grow and cannot be expanded or easily removed. Cartilage grafts, which can replace the weaker area of the trachea, are also rarely used (44). In addition, several projects using 3D printing of the trachea have been undertaken (45), though few have yielded results consistent with acceptable clinical standards.

## Postoperative Follow-Up

The fact that bronchoscopy early in the postoperative period may not reveal a significantly different appearance of the trachea cannot be overemphasized. Similarly, in some children, more time is required for symptom improvement. Patience is paramount and follow-up encompassing observation of clinical improvement and serial bronchoscopy or CT scans is essential. Some patients may present severe tracheomalacia on exams, but have virtually no signs and symptoms.

After surgical intervention, tracheomalacia is considered clinically significant when a patient has recurrent pneumonia, recurrent hospitalizations for airway issues, or persistent cyanotic spells. In this setting, revision surgery may be necessary.

## Conclusion

Cyanotic spells represent a potentially life-threatening condition. For patients with OA and TOF that was previously repaired, the possibility of a recurrent or missed TOF should be eliminated. Primary etiologies of cyanotic spells include severe tracheomalacia and innominate artery compression. Investigations should minimally include an endoscopic exam with flexible and/or rigid bronchoscopy. Imaging studies such as MRI with contrast can help to determine mediastinal anatomy and the presence of a vascular anomaly. Multiple medical and surgical options exist and must be promptly initiated to avoid serious consequences of these cyanotic spells.

## Author Contributions

MB, AC, and RC: writing, revision, editing, and review of literature.

## Conflict of Interest Statement

The authors declare that the research was conducted in the absence of any commercial or financial relationships that could be construed as a potential conflict of interest. The reviewer, MS, and handling editor declared their shared affiliation, and the handling editor states that the process nevertheless met the standards of a fair and objective review.
